# From Exhaustion to Disengagement via Self-Efficacy Change: Findings from Two Longitudinal Studies among Human Services Workers

**DOI:** 10.3389/fpsyg.2015.02032

**Published:** 2016-01-08

**Authors:** Anna Rogala, Kotaro Shoji, Aleksandra Luszczynska, Anna Kuna, Carolyn Yeager, Charles C. Benight, Roman Cieslak

**Affiliations:** ^1^Department of Psychology, Szkoła Wyższa Psychologii Społeczne University of Social Sciences and HumanitiesWarsaw, Poland; ^2^Department of Psychology, Trauma Health and Hazards Center, University of Colorado Colorado SpringsColorado Springs, CO, USA; ^3^Wroclaw Department, Szkola Wyzsza Psychologii Spoleczne University of Social Sciences and HumanitiesWroclaw, Poland

**Keywords:** burnout, exhaustion, disengagement, self-efficacy, social support

## Abstract

This longitudinal research examined the relationship direction between burnout components (exhaustion and disengagement) within the context of personal resources measured by self-efficacy and social support. In line with the conservation of resources theory we hypothesized that exhaustion may trigger a spiral loss of personal resources where self-efficacy declines and subsequently, social support also declines and in turn predict disengagement. Participants in Study 1 were mental healthcare providers (*N* = 135) working with U.S. military personnel suffering from trauma. Participants in Study 2 were healthcare providers, social workers, and other human services professionals (*N* = 193) providing various types of services for civilian trauma survivors in Poland. Baseline and 6-month follow-up measurements included burnout components, burnout self-efficacy and perceived social support. The path analysis showed consistent results for both longitudinal studies; exhaustion measured at Time 1 led to disengagement at Time 2, after controlling for baseline disengagement levels. Across Study 1 and Study 2 these associations were mediated by self-efficacy change: Higher exhaustion led to greater decline in self-efficacy which in turn explained higher disengagement at the follow-up. Social support, however, did not mediate between self-efficacy and disengagement. These mediating effects were invariant across Studies 1 and 2, although the mean levels of burnout and personal resources differed significantly. The results contribute to a discussion on the internal structure of job burnout and a broader understanding of the associations between exhaustion and disengagement that may be explained by the underlying mechanism of change in self-efficacy.

## Introduction

Job burnout is recognized as one of the key consequences of job stress (Maslach et al., 2001). Its high prevalence was demonstrated across occupational groups of human services workers, reaching up to 67% for burnout in a community of mental health workers (Morse et al., 2012). Predictors of burnout and co-occurring mental health problems have been thoroughly investigated (Maslach and Leiter, 2008; Leiter et al., 2013; Cieslak et al., 2014). However, only a few studies have examined the causal relationships among burnout components (Taris et al., 2005). Our study aims to fill this gap by examining the effects of exhaustion on disengagement, two core components of burnout. The effects of exhaustion on disengagement will be evaluated further in the context of potential indirect pathways through personal resources (via self-efficacy and social support; Schwarzer and Knoll, 2007).

Traditionally, burnout has been conceptualized as a prolonged response to chronic emotional and interpersonal stressors that occur in the work setting (Maslach et al., 2001). The three original components of burnout proposed by Maslach et al. (2001) are (1) emotional exhaustion; the feeling of being overstrained and depleted of emotional and physical resources, (2) depersonalization; a negative and cynical attitude toward people, and (3) reduced personal accomplishment; the tendency to evaluate one's work negatively and diminish one's own achievements. In the process of generalizing burnout to processes observed in occupations other than human services the original names of the burnout components were changed (Maslach et al., 2001). In particular, emotional exhaustion became exhaustion, depersonalization was replaced with cynicism, and reduced personal accomplishment was replaced with a lack of professional efficacy. Research confirmed that the three components of burnout were interrelated (Lee and Ashforth, 1996; Taris et al., 2005; Houkes et al., 2011).

Across several models of burnout, exhaustion is one of its key facets. For example Melamed et al. (2006) viewed burnout as a multidimensional construct consisting of emotional exhaustion, physical fatigue, and cognitive weariness. Other approaches suggested that burnout might be reduced to a single common experience of exhaustion (cf. Kristensen et al., 2005; Malach-Pines, 2005).

Yet another prominent model of burnout (Demerouti et al., 2001) assumed only two components, exhaustion and disengagement. In this model exhaustion accounts not only for affective, but also physical and cognitive aspects (Demerouti et al., 2001). Disengagement refers to both withdrawing oneself from work and creating negative attitudes toward one's work. Thus, disengagement is broader than depersonalization in that it refers to emotions toward the work as well as the relational elements such as engagement in work tasks or identification with one's work (Demerouti et al., 2001). Following this new conceptualization of exhaustion and disengagement, an alternative measure of job burnout was proposed (cf. Oldenburg Burnout Inventory; OLBI; Halbesleben and Demerouti, 2005).

Dropping the personal accomplishment component is in line with theoretical developments (cf. Demerouti et al., 2001) and meta-analyses pointing out that this concept may be difficult to distinguish from other constructs, such as self-efficacy (Cordes and Dougherty, 1993; Shoji et al., 2015). It has also been argued that the personal accomplishment component develops to a great extent independently from exhaustion and disengagement (Lee and Ashforth, 1993; Taris et al., 2005). Importantly, the independence of self-efficacy and personal accomplishment may be questionable (Cordes and Dougherty, 1993). A recent meta-analysis of 57 studies confirmed that compared to other burnout dimensions, personal accomplishment forms the strongest relationship with self-efficacy (Shoji et al., 2015).

Understanding the interplay between burnout components is also critically important to consider. Three different models have been proposed. First, Demerouti et al. (2001) indicated that exhaustion is a consequence of prolonged physical, affective, and cognitive work stress. Although, Demerouti et al. (2001) suggested that exhaustion and disengagement are not causally related, they assumed that exhaustion may develop faster than disengagement because of higher individual sensitivity to job demands. In contrast, Leiter and Maslach (1988) argued in their process model that chronic job stress leads to emotional exhaustion, which in turn causes depersonalization. These prolonged feelings of depersonalization may in turn result in reduced personal accomplishment. In comparison, Lee and Ashforth (1993) offered a different approach. In line with the process model, they suggested that depersonalization may result from emotional exhaustion. However, in contrast to Leiter and Maslach (1988), they argued that reduced personal accomplishment is derived from a heightened level of emotional exhaustion rather than depersonalization. The third model proposed suggested a phase approach (cf. Taris et al., 2005). The phase model begins with depersonalization in response to heightened work stress leading to negative beliefs about one's achievements. Emotional exhaustion then follows due to high depersonalization levels and low personal accomplishment beliefs.

Collectively these models proposed different directions for the relationships between burnout components. All three models suggested that exhaustion is a response to work stress (Leiter and Maslach, 1988; Lee and Ashforth, 1993; Demerouti et al., 2001) and two of the models (Leiter and Maslach, 1988; Lee and Ashforth, 1993) argued that exhaustion causes depersonalization.

There are a limited number of longitudinal studies investigating the direction of associations among job burnout components, yet the findings are relatively consistent. In support of the process model (Leiter and Maslach, 1988), Taris et al. (2005) found that exhaustion predicted depersonalization, which in turn predicted lack of accomplishments. Another longitudinal investigation (Diestel and Schmidt, 2010) indicated that exhaustion predicted depersonalization and that both exhaustion and depersonalization explained personal accomplishment, measured at a 12-month follow-up. A study with an 8-year follow-up provided evidence that exhaustion predicted cynicism (Toppinen-Tanner et al., 2002). Houkes et al. (2011) found that among women, emotional exhaustion triggered depersonalization, which in turn predicted reduced personal accomplishment. Among men, however, depersonalization preceded exhaustion (Houkes et al., 2011). This differential gender effect may result from differences in gender-related individual characteristics and differences in working conditions among men and women. For instance, Houkes et al. (2011) suggested that women may face more challenges in the areas of work-life balance, which may cause more emotional exhaustion among women. Men may use avoidance coping strategies more frequently than women. Depersonalization may reflect the use of avoidance coping (disengagement) and, therefore, depersonalization may be more salient among men (Houkes et al., 2011). In sum, the majority of (albeit not all) the research suggested that exhaustion precedes depersonalization. Importantly, none of these longitudinal studies evaluated the underlying mediating mechanisms that may explain why exhaustion might lead to disengagement or cynicism. The present studies attempt to fill this void.

Self-efficacy and social support are among the most frequently examined resources that play important roles in understanding the development of work stress consequences such as burnout (Cordes and Dougherty, 1993; Perrewe et al., 2002; Smoktunowicz et al., 2015). Perceived social support represents the perception that help provided by others (e.g., coworkers, supervisors) is adequate and also refers to the perceived quality of support which facilitates adjustment (Schwarzer and Knoll, 2007). Self-efficacy is defined as one's beliefs in one's own ability to manage environmental demands and exercise control over one's own functioning (Bandura, 1997). According to social cognitive theory (Bandura, 1997; Luszczynska and Schwarzer, 2015) self-efficacy measures should be context-specific because self-efficacy itself is a context-specific belief. Self-efficacy measures applied in the context of burnout account for a broad range of work-related competences. For example, they cover workers' confidence that they can employ the skills necessary to deal with job-specific tasks, and ability to cope with job-specific challenges (Shoji et al., 2015). Yet, the systematic review by Shoji et al. (2015) did not identify a measure of self-efficacy that focused on these aspects of burnout. Previous research on burnout suggests developing and applying context-specific self-efficacy measures, because they better predict burnout and work-stress related outcomes (Salanova et al., 2002). Therefore, to gain a better insight into the relationships between burnout components research should apply a measure of self-efficacy focusing on dealing with burnout-related issues.

Theoretical models explaining burnout consistently propose that control beliefs (including self-efficacy) and social support constitute critical resources that are important to consider (cf. job demands-control-support [DCS] model, Karasek and Theorell, 1990; the conservation of resources [COR] theory; Hobfoll, 1989). Low levels of these resources lead to negative individual and organizational consequences, such as exhaustion and depersonalization (Karasek and Theorell, 1990). Cross-sectional and longitudinal research has confirmed that baseline levels of self-efficacy and social support independently explain disengagement (or engagement) and exhaustion (Llorens et al., 2007; Xanthopoulou et al., 2007; Huynh et al., 2013; Yu et al., 2015). The key limitations of models such as DCS is that resources are depicted as static. Consequently, studies inspired by these models examined baseline levels of resources rather than changes in resources. A notable exception is COR theory that takes a dynamic approach including resource change as a central mechanism (Hobfoll, 1989; Hobfoll et al., 2003).

COR theory (Hobfoll, 1989, 2011) suggests that a loss or depletion of a broad range of resources (e.g., emotional exhaustion and reduced motivation to engage in various challenging tasks) may cause further loss of personal resources (such as self-efficacy and social support). Hobfoll refers to this as a loss spiral. For example, the state of exhaustion may be used as a starting point for depicting the loss sequence between burnout and resources. Exhaustion can be viewed as one facet of resource depletion. The subsequent loss of personal resources in turn increases the likelihood of developing specific negative consequences (Hobfoll, 1989, 2011). Thus, it may be assumed that a high level of exhaustion captures a stage in the loss spiral that is followed by further losses of personal resources such as self-efficacy or perceived social support. Importantly, resources operate in sequence as “caravans” (Hobfoll, 2011), not as independent factors. People who exhaust their resources are most vulnerable to additional losses that lead to a further depletion of their resources (Hobfoll, 1989, 2011). Concluding, it may be assumed that exhaustion may be a precursor to a further loss of resources, causing a negative change in self-efficacy and a decline in perceived social support. This, in turn, would increase the likelihood of other negative consequences of work stress, such as disengagement (which may develop to prevent further loss of personal resources), absenteeism, and turnover.

We found one longitudinal study confirming that emotional exhaustion has an effect on self-efficacy and that self-efficacy may mediate the relationship between exhaustion and other burnout components (Brouwers and Tomic, 2000). A possible explanation of this effect is related to two sources of self-efficacy, namely mastery experiences and somatic/emotional states (Brouwers and Tomic, 2000). High levels of emotional exhaustion may lead to a reduction in mastery experiences. Moreover, aversive somatic and emotional arousal connected with exhaustion theoretically would also result in reduced self-efficacy beliefs (Bandura, 1997).

As suggested by Hobfoll (2011), resources do not operate in a parallel manner but rather they form “a caravan.” Schwarzer and Knoll (2007) linked self-efficacy and social support by proposing the cultivation hypothesis. People with a higher levels of self-efficacy are more effective in finding, maintaining, and developing supportive social relationships, therefore, social support is maintained by self-efficacy. An alternative enabling hypothesis suggests that social support facilitates self-efficacy (Schwarzer and Knoll, 2007). Research conducted in the context of secondary traumatization among human services workers provided support for the cultivation hypothesis, but not for the enabling hypothesis (Shoji et al., 2014).

Our studies investigated the associations between two components of burnout, exhaustion and disengagement within the context of personal resources. We investigated the importance of changes in two primary personal resources, burnout self-efficacy and work related social support. The associations were tested in two longitudinal studies conducted among human services workers working in the U.S. and Poland with military and civilian clients. Specifically, it was hypothesized that exhaustion at Time 1 would predict disengagement at Time 2. Second, we hypothesized that the exhaustion—disengagement association would be sequentially mediated by changes in self-efficacy and changes in social support. These mediating effects were tested after controlling for Time 1 disengagement. The hypotheses were tested controlling for years of work experience. This variable is one of the key determinants of burnout (Brewer and Shapard, 2004), producing similar effects across different cultures (Gill et al., 2012).

## Study 1

### Methods

#### Participants

Study 1 was a part of a larger study investigating secondary traumatic stress and job burnout among behavioral healthcare providers for U.S. military personnel. Inclusion criteria for this study included (a) working as a behavioral healthcare provider at least one year, (b) providing services for U.S. military personnel, and (c) being indirectly exposed to trauma through their work. Two hundred and ninety four participants (mean age = 48.87 years old [*SD* = 12.76], 66.3% women) completed the online survey at Time 1 (T1). Among those, 135 participants (mean age = 50.62 years old [*SD* = 12.58], 71.1% women) completed the online survey at Time 2 (T2). Table 1 displays demographic information for completers (*n* = 135). At Time 1, participants reported various indirect traumatic experiences (i.e., secondary exposure through their work), including life-threatening illness or injury of a client or someone close (91.9%), combat exposure (91.1%), sudden unexpected death of someone close (90.4%), sexual assault (87.4%), physical assault (85.9%), transportation accidents (83.7%), natural disasters (68.9%), other serious accidents (63.7%), and other life threatening crimes (57.0%).

**Table 1:** **Descriptive statistics for demographics for Study 1 (U.S. Data) and Study 2 (Polish Data)**.

**Measure**	**Study 1: Time 1**	**Study 1: Time 2**	**Study 2: Time 1**	**Study 2: Time 2**
Mean age in years (*SD*)	48.87 (12.76)	50.62 (12.58)	35.32 (8.48)	34.97 (8.06)
**GENDER**				
Female	66.3% (195)	71.1% (96)	76.1% (233)	79.3% (153)
Male	33.7% (99)	28.9% (39)	22.9% (70)	19.2% (37)
**INTIMATE RELATIONSHIP**				
In a long-term relationship	76.2% (224)	72.6% (98)	73.9% (226)	77.7% (150)
Not in a long term relationship	21.4% (63)	25.2% (34)	25.5% (78)	21.8% (37)
**HIGHEST DEGREE**				
High school	0.3% (1)	0 (0%)	20.6% (63)	18.1% (35)
Associate's degree	0.3% (1)	0 (0%)	−	−
Bachelor's degree	2.0% (6)	1.5% (2)	21.6% (66)	20.2% (39)
Master's degree	45.2% (133)	51.1% (69)	56.6% (172)	60.1% (116)
Doctorate degree	52.0% (153)	47.4% (64)	1.0% (3)	0.5% (1)
**PROFESSION**				
	116 CP (39.5%)	50 CP (37.0%)	148 HCP (48.4%)	89 HCP (46.1%)
	74 counselors (25.2%)	39 counselors (28.9%)	115 SW (37.6%)	78 SW (40.4%)
	57 SW (19.4%)	28 SW (20.7%)	38 others (12.4%)	23 others (11.9%)
	28 HCP (9.5%)	9 HCP (6.7%)		

*Sample size for Study 1 was 294 for Time 1 and 135 for Time 2. Sample size for Study 2 for Time 1 was 306 and 193 for Time 2. Some percentages did not add up to 100% because of missing data. Long-term relationship included married couples and couples in a committed relationship. CP, clinical psychologist; HCP, health care provider; SW, social worker*.

#### Measurement

Participants completed questionnaires assessing job burnout, self-efficacy for job burnout, social support, and demographics.

#### Burnout

Oldenburg Burnout Inventory (OLBI; Halbesleben and Demerouti, 2005) was used to assess emotional exhaustion and disengagement. Respondents rated the agreeableness for each statement regarding work-related distress on a 5-point scale ranging from 1 (*strongly disagree*) to 5 (*strongly agree*). Sample items included “Lately, I tend to think less at work and do my job almost mechanically” for the disengagement subscale and “After work, I tend to need more time than in the past in order to relax and feel better” for the emotional exhaustion subscale. Cronbach's alpha coefficients were 0.86 for disengagement at T1 and T2, 0.81 for exhaustion at T1, and 0.86 for exhaustion at T2.

#### Change in burnout-related self-efficacy

An 11-item Burnout Self-Efficacy Scale was applied to measure self-efficacy for dealing with job burnout (Lua, 2008). The scale was developed in line with Bandura's (1997) suggestion to construct self-efficacy measures with all items reflecting specific demands facing human services workers dealing with negative consequences of work stress. The initial research conducted among 252 Singaporean employees showed good reliability of the scale, with Cronbach's alpha of 0.93, and good discriminant validity, as shown by moderate correlations with other resource and burnout indicators (Lua, 2008). Each question begins with the stem “How capable am I to…,” followed with items such as “deal with a feeling that this job wears me out” and “handle the feeling that my job is useless.” The responses ranged from 1 (*very incapable*) to 7 (*very capable*). In the present study Cronbach's alpha coefficient was 0.91 at T1 and T2. Standardized residual values were used as the index of change. To obtain the standardized residual values T2 self-efficacy was entered in the regression analysis as a dependent variable and T1 self-efficacy was entered as a predictor. A higher value of the index means a higher increase of self-efficacy whereas a lower value means a greater decrease of self-efficacy. A similar approach was used previously (Benight et al., 2008).

#### Change in perceived social support

The Multidimensional Scale of Perceived Social Support (MSPSS; Zimet et al., 1988) was used to assess perceived social support. Respondents rated the agreeableness for each statement regarding their perception of social support from family, friends, and significant others on a 7-point scale ranging from 1 (*very strongly disagree*) to 7 (*very strongly agree*). The original instruction was modified to reflect support from family, friends, and significant others (including co-workers, and supervisors), enabling participants to cope with difficulties at work. Sample items included “I can talk about my problems with my family,” “My friends really try to help me,” and “There is a special person who is around when I am in need.” Cronbach's alpha coefficient was 0.94 at T1 and T2. Similar to self-efficacy for job burnout, residual change scores were calculated as the index of change.

#### Demographics

We collected demographic information regarding the number of years of work experience, participants' age, gender, level of education, relationship status, occupation, and experiences with indirect exposure to traumatic events through their work.

#### Procedures

The Institutional Review Board at the authors' institution in the U.S. approved this study. The details of the procedures were described elsewhere (Cieslak et al., 2013a; Shoji et al., 2014). Potential participants received the invitation email containing the online survey link. They indicated whether they agreed to participate in the study on the online informed consent form. Those who agreed to take part in T2 assessment received the invitation email with the online survey link 6 months after the T1 survey. The mean time elapsed between T1 and T2 was 195.77 days (*SD* = 20.00).

#### Analytical procedures

We used the maximum likelihood estimation method to impute missing data for 135 completers using IBM SPSS Amos (version 22). Measurement items for burnout, change in social support and self-efficacy were included in the full information maximum likelihood imputation. The assumption of this approach to data imputation is that the missing data must be missing at random (MAR). To assess MAR, Little's missing completely at random (MCAR) tests, which is more restrictive than MAR, were conducted in IBM SPSS (version 22) using gender, profession, and intimate relationship status as references. Results of the Little's MCAR tests showed missing data were MCAR for items for MSPSS at T1, $\chi_{\left(12\right)}^{2}=8.09$, *p* = 0.78, items for self-efficacy for job burnout at T1, $\chi_{\left(11\right)}^{2}=8.75$, *p* = 0.65, items for OLBI at T1, $\chi_{\left(30\right)}^{2}=32.67$, *p* = 0.34, items for MSPSS at T2, $\chi_{\left(12\right)}^{2}=8.87$, *p* = 0.71, items for self-efficacy for job burnout at T2, $\chi_{\left(12\right)}^{2}=2.29$, *p* = 0.99, and items for OLBI at T2, $\chi_{\left(12\right)}^{2}=27.37$, *p* = 0.39. In total, 0.08% of missing data were replaced (0.04% at Time 1 and 0.11% at Time 2). Mardia's coefficient indicated a slight deviation from multivariate non-normality (critical ratio of 3.43). We examined bootstrap confidence intervals of coefficients for consistency of results.

**Figure 1:**
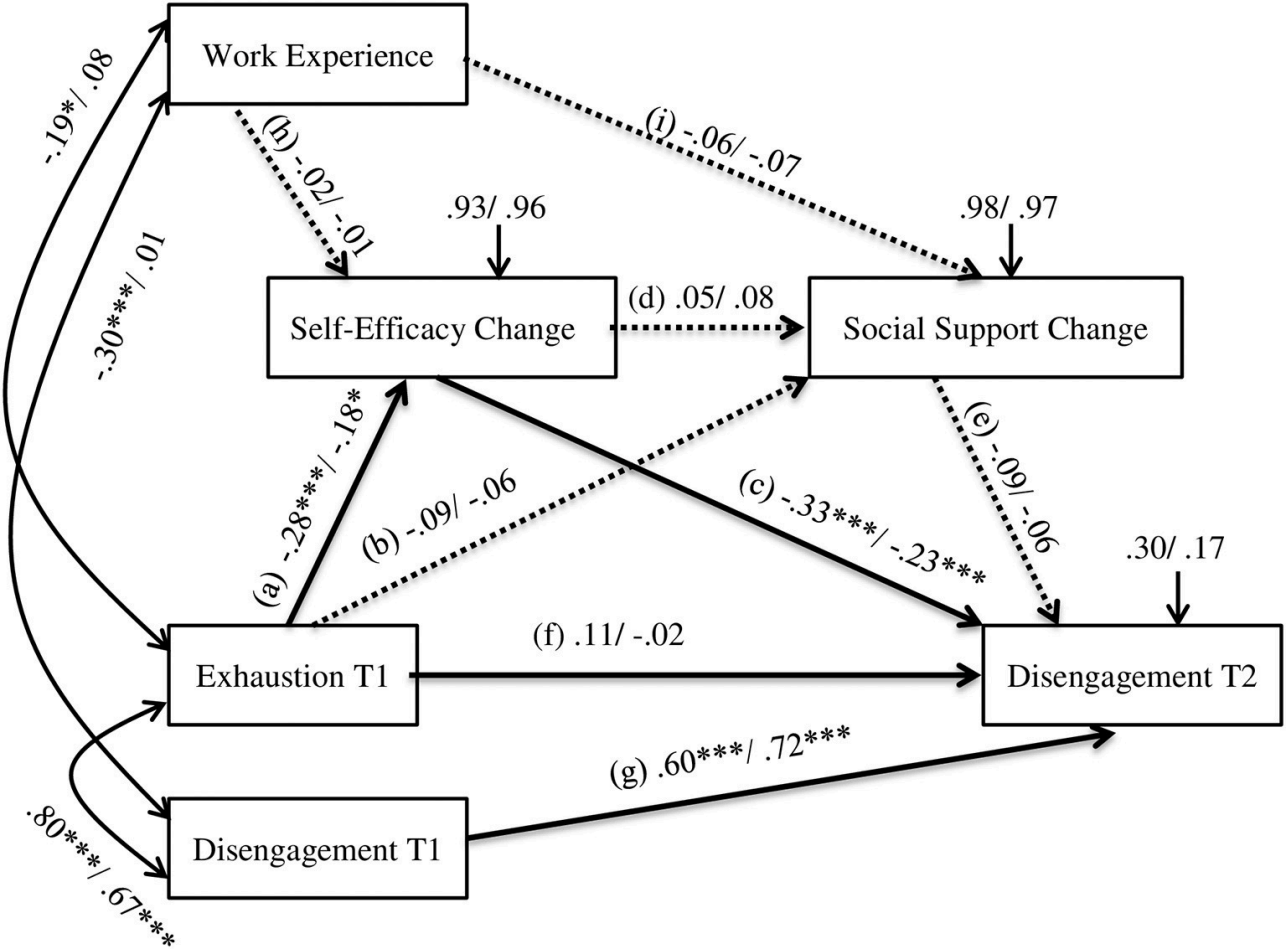
**Cultivation model with the sequential mediation effect of self-efficacy change and social support change in the relationship between exhaustion and disengagement**. Values before the slash indicate standardized coefficients for Study 1. Values after the slash indicate standardized coefficients for Study 2. Dotted arrows indicate the pathways constrained to be zero in the analysis of nested models. Coefficients for unconstrained model in the test of invariance: **(A)** −0.28/−0.18, **(B)** −0.09/−0.06, **(C)** −0.32/−0.02, **(D)** 0.05/0.08, **(E)** −0.09/−0.06, **(F)** 0.10/−0.02, **(G)** 0.61/0.72, **(H)** −0.02/−0.01, **(I)** −0.08/−0.06. Coefficients for the final model with significant pathways and residuals constrained to be equal: **(A)** −0.22/−0.22, **(B)** −0.09/−0.06, **(C)** −0.25/−0.28, **(D)** 0.05/0.08, **(E)** −0.10/−0.06, **(F)** 0.02/0.02, **(G)** 0.71/0.66, **(H)** −0.01/−0.01, **(I)** −0.08/−0.06. ^***^P < 0.001, ^*^P < 0.05.

We tested the cultivation hypothesis in the sequential mediation analysis using Mplus (see Figure 1). We used exhaustion at T1 as the independent variable and disengagement at T2 as the dependent variable. Disengagement at T1 and years of work experience were used as covariates. In this model, the relationship between exhaustion at T1 and disengagement at T2 was sequentially mediated by the change index of self-efficacy and the change index of social support. Each indirect effect was tested using 95% bootstrap confidence intervals with 10,000 bootstrap samples. We used three fit indices to assess model-data fit. We used a cutoff point <0.10 for the root mean square error of approximation (RMSEA; Browne and Cudeck, 1993), a cutoff point >0.90 for the comparative fit index (CFI; Hu and Bentler, 1999), and a cutoff point <0.08 for the standardized root mean residual (SRMR; Hu and Bentler, 1999).

### Results

Table 1 depicts the descriptive statistics for the demographic variables. Table 2 displays correlation coefficients, means, and standard deviations for the study variables. Attrition analyses showed that dropouts had significantly higher disengagement at T1 than did completers, *t*_(292)_ = 2.51, *p* = 0.01, and completers were significantly older than dropouts, *t*_(288)_ = 2.17, *p* = 0.03. There were no significant differences between dropouts and completers in emotional exhaustion at T1, *t*_(292)_ = 1.87, *p* = 0.06, self-efficacy at T1, *t*_(292)_ = 1.15, *p* = 0.25, social support at T1, *t*_(292)_ = 0.30, *p* = 0.70, gender, $\chi_{\left(1\right)}^{2}=$ 2.56, *p* = 0.11, profession, $\chi_{\left(3\right)}^{2}=$ 4.12, *p* = 0.25, intimate relationship status, $\chi_{\left(1\right)}^{2}=$ 2.07, *p* = 0.15, or education, $\chi_{\left(4\right)}^{2}=$ 5.01, *p* = 0.29. Women and men did not differ significantly across the study variables (*p*-value range: 0.14–0.88).

**Table 2:** **Means, standard deviations, pearson's correlations among study variables for Study 1 (below the diagonal) and Study 2 (above the diagonal), and comparisons between data from two studies (Time 2)**.

										**Mean (*SD*)**				
**Measure**	**1**	**2**	**3**	**4**	**5**	**6**	**7**	**8**	**9**	**Study 1**	**Study 2**	***t***	***d***	**95% CIs**
1. Disengagement T1		0.74^***^	0.67^***^	0.57^***^	−0.61^***^	−0.58^***^	−0.19^***^	−0.18^*^	0.01	2.35 (0.70)	2.71 (0.64)	4.92	0.53	0.22–0.51
2. Disengagement T2	0.77^***^		0.49^***^	0.66^***^	−0.45^***^	−0.58^***^	−0.12	−0.19^**^	−0.05	2.40 (0.75)	2.77 (0.65)	3.64	0.53	0.13–0.43
3. Exhaustion T1	0.80^***^	0.67^***^		0.69^***^	−0.69^***^	−0.61^***^	−0.22^***^	−0.15^*^	0.08	2.54 (0.70)	2.82 (0.69)	4.72	0.40	0.21–0.52
4. Exhaustion T2	0.64^***^	0.76^***^	0.77^***^		−0.57^***^	−0.60^***^	−0.21^**^	−0.22^**^	0.06	2.53 (0.76)	2.81 (0.60)	3.51	0.41	0.13–0.42
5. Self-efficacy T1	−0.58^***^	−0.35^***^	−0.52^***^	−0.38^***^		0.70^***^	0.23^***^	0.22^**^	−0.04	5.89 (0.89)	5.24 (0.98)	6.26	0.69	0.44–0.86
6. Self-efficacy T2	−0.55^***^	−0.62^***^	−0.52^***^	−0.61^***^	0.56^***^		0.23^***^	0.24^***^	−0.05	5.94 (0.81)	5.22 (0.95)	7.31	0.82	0.52–0.91
7. Social support T1	−0.32^***^	−0.31^***^	−0.29^***^	−0.23^**^	0.21^*^	0.17		0.31^***^	0.03	5.80 (1.08)	4.93 (1.58)	5.99	0.64	0.57–1.18
8. Social support T2	−0.29^***^	−0.34^***^	−0.29^***^	−0.30^***^	0.18^*^	0.1^*^	0.82^***^		−0.06	5.73 (1.16)	5.12 (1.36)	4.37	0.48	0.33–0.89
9. Work experience T1	−0.30^***^	−0.27^***^	−0.19^*^	−0.23^**^	0.34^***^	0.22^*^	0.03	−0.01		17.07 (11.36)	12.92 (9.18)	3.52	0.40	1.92–6.39

*Correlations in the lower diagonal region show values for U.S. data (Study 1). Correlations in the upper diagonal region show values for Polish data (Study 2). t-tests were conducted for each variable between Study 1 and Study 2. Sample sizes were 135 for Study 1 and 193 for Study 2. T1, Time 1; T2, Time 2. All t-scores were significant at p < 0.001*.

*** *p < 0.001*,

** *p < 0.1*,

* *p < 0.05*.

Repeated measures analysis of variance indicated that burnout self-efficacy did not change significantly from T1 to T2, *F*_(1, 134)_ = 0.44, *p* = 0.509, η^2^ = 0.003. Similarly, the levels of perceived social support remained similar across the measurement points, *F*_(1, 134)_ = 1.63, *p* = 0.205, η^2^ = 0.012. In line with those findings a correlation analysis indicated relatively high stability across study variables, with T1 and T2 correlation coefficients ranging from 0.56 (self-efficacy) to 0.82 (social support). High correlations between T1 and T2 indicators of self-efficacy and social support may result in low variance of raw change scores and, therefore, limit the usefulness of such scores. Instead of raw change scores we applied residualized change scores. Residualized scores are weakly associated with T1 scores and maintain high reliability even when the correlations between T1 and T2 are high (Allison, 1990).

The examination of the hypothesized model assuming a sequential mediation effect of self-efficacy change and social support change in the relationship between exhaustion at T1 and disengagement at T2 showed that the model had adequate model-data fit, RMSEA = 0.032 (90% CI [0.000, 0.152]), CFI = 0.997, Tucker-Lewis Index (TLI; Tucker and Lewis, 1973) = 0.990, SRMR = 0.018. Figure 1 shows standardized coefficients for each parameter in the model. Bootstrap confidence intervals and *p* values were consistent. Based on bootstrap confidence intervals, the pathway through the residuals of self-efficacy is significant (95% bootstrap CI [0.035, 0.187]). However, neither the pathways through the social support change (95% bootstrap CI [−0.016, 0.032]) nor the pathways through the self-efficacy change and subsequently social support change (95% bootstrap CI [-0.004, 0.006]) were significant. These results indicated that high levels of exhaustion at T1 predicted a greater decrease in self-efficacy, which in turn led to higher levels of disengagement at T2.

Additionally, we tested a nested model with nonsignificant pathways (from self-efficacy change to social support change, from T1 exhaustion to social support change, and from social support change to T2 disengagement) constrained to zero. Results showed that the nested model was not significantly different from the hypothesized model, $\chi_{\left(3\right)}^{2}$ = 4.79, *p* = 0.19. Thus, this nested model may be accepted.

### Discussion

The results of Study 1 did not support the cultivation hypothesis among behavioral healthcare providers working for U.S. military personnel. However, we found an indirect effect of a decline in self-efficacy in the relationship between exhaustion at T1 and disengagement at T2. In Study 2, to replicate these findings, the same model was tested among Polish professionals working with people suffering from an exposure to traumatic events.

## Study 2

### Methods

#### Participants

Study 2 was a part of larger study examining work-related resources and demands among human services professionals who were indirectly exposed to traumatic events through their work. Inclusion criteria for this study were (a) working at least 1 year as a healthcare or social service provider, (b) providing services for civilians who were exposed to traumatic events, and (c) indirectly experiencing traumatic events through their work. Three hundred and six participants (mean age = 35.32 [*SD* = 8.48], 76.1% women) completed the online survey at T1. Of those, 193 participants (mean age = 34.97 [*SD* = 8.06], 79.3% women) completed the online survey at T2. Table 1 displays demographics for 193 completers for Study 2. At T1, they were exposed to a number of indirect traumatic events, including illness or injury to clients or loved one (89.6%), physical violence (88.1%), sudden unexpected death of loved one (84.5%), transportation accidents (72.0%), other serious accidents (64.2%), natural disasters (31.1%), sexual violence (51.3%), other serious life threatening crime (39.9%), combat (7.3%), other traumatic events (35.2%).

#### Measurement

We used the Polish version of the same measurements as in Study 1 to assess burnout, self-efficacy changes, social support changes, and demographics. Back translation was used to establish accurate translation from English to Polish. Cronbach's alpha coefficients were 0.80 for disengagement at T1,0.81 for disengagement at T1,0.83 for exhaustion at T1,0.78 for exhaustion at T2,0.91 for self-efficacy at T1 and T2, and.96 for MSPSS at T1 and T2. As in Study 1, residuals between self-efficacy at T1 and T2 and residuals between social support at T1 and T2 were used as the change indices.

#### Procedures

The Internal Review Board at the authors' institution in Poland approved this study. The details of the procedures were described elsewhere (Cieslak et al., 2013a,b; Shoji et al., 2014). The message for invitation to the study was posted on social networking websites for professionals who were potentially exposed to indirect traumatic events. After completion of the T1 assessment, those who agreed to take part in the T2 assessment received the invitation email. The mean time elapsed between T1 and T2 was 162.12 days (*SD* = 39.39).

#### Analytical procedures

The same analytical procedures and software were used as in Study 1 on 193 completers of the study. The Little's MCAR tests showed that missing data were MCAR for items for OLBI at T1, $\chi_{\left(76\right)}^{2}=119.44$, *p* = 0.45, items for MSPSS at T1, $\chi_{\left(76\right)}^{2}=89.69$, *p* = 0.14, items for self-efficacy for job burnout at T1, $\chi_{\left(64\right)}^{2}=61.99$, *p* = 0.55, items for OLBI at T2, $\chi_{\left(136\right)}^{2}=161.14$, *p* = 0.07, and items for MSPSS at T2, $\chi_{\left(55\right)}^{2}=55.73$, *p* = 0.45. However, items for self-efficacy for job burnout at T2 were not MCAR, $\chi_{\left(76\right)}^{2}=112.18$, *p* < 0.01. Items for self-efficacy at T2 contained only 0.22% of missing data; therefore, these items were imputed with other missing data. In total 0.56% of data (0.52% at Time 1, 0.62% at Time 2) were imputed as missing data. The same analytic approach was utilized as in Study 1 to test our primary hypotheses. Mardia's coefficient indicated a slight deviation from multivariate non-normality (critical ratio of 4.39). We examined the consistency between significance of coefficients and 95% bootstrap confidence intervals.

### Results

Table 2 displays means, standard deviations, and Pearson correlations for 193 participants for Study 2. Attrition analysis showed that there were no significant differences between completers and dropouts in disengagement at T1, *t*_(304)_ = 1.07, *p* = 0.29, exhaustion at T1, *t*_(304)_ = 0.16, *p* = 0.87, self-efficacy at T1, *t*_(304)_ = 0.44, *p* = 0.66, social support at T1, *t*_(304)_ = 0.92, *p* = 0.36, age, *t*_(304)_ = 0.92, *p* = 0.36, gender, $\chi_{\left(1\right)}^{2}=3.78$, *p* = 0.05, profession, $\chi_{\left(2\right)}^{2}$ = 1.77, *p* = 0.41, and education, $\chi_{\left(3\right)}^{2}=$ 4.51, *p* = 0.21. Those who were in a long-term relationship tended to dropout more frequently, $\chi_{\left(1\right)}^{2}\;=$ 3.91, *p* = 0.05. Emotional exhaustion at T1 was significantly higher among women (*M* = 2.86) than among men (*M* = 2.59), *t*_(188)_ = 2.17, *p* = 0.03. Women reported significantly higher social support at T2 (*M* = 5.23), compared to men (*M* = 4.66), *t*_(188)_ = 2.31, *p* = 0.02.

Repeated measures analysis of variance showed that burnout self-efficacy did not change from T1 to T2, *F*_(1, 192)_ = 0.10, *p* = 0.752, η^2^ = 0.001. The levels of perceived social support remained similar across the measurement points, *F*_(1, 192)_ = 2.35, *p* = 0.127, η^2^ = 0.127. Correlation analysis confirmed moderate-to-high stability across study variables, with T1 and T2 correlation coefficients ranging from 0.23 (social support) and 0.31 (self-efficacy), to 0.74 (disengagement). As in Study 1, we applied residualized change scores as the indicators of change in self-efficacy and social support.

Results of the sequential mediation analysis of self-efficacy change and social support change in the relationship between exhaustion at T1 and disengagement at T2 showed that the model had adequate fit, RMSEA = 0.080 (90% CI [0.000, 0.162]), CFI = 0.980, TLI = 0.919, SRMR = 0.034. Significance levels of all coefficients were consistent with results of 95% bootstrap confidence intervals. Figure 1 displays standardized coefficients for parameters in the model. Bootstrap confidence intervals indicated that the pathway through self-efficacy change was significant (95% bootstrap CI [0.004, 0.079]). The pathway through social support changes (95% bootstrap CI [−0.003, 0.024]) as well as the pathway through self-efficacy change and social support change (95% bootstrap CI [0.000, 0.007]) were not significant.

As in Study 1, a nested model with constraints and the hypothesized model with no constraints were compared. In the nested model, three pathways were constrained to zero: from self-efficacy change to social support change, from social support change to T2 disengagement, and from T1 exhaustion to social support change. Results indicated that the nested model was not significantly different from the hypothesized model, $\chi_{\left(3\right)}^{2}=$ 3.96, *p* = 0.27, therefore it may be accepted.

#### Test of invariance of associations across study 1 and study 2

The invariance of the findings across the two studies was tested using a two-group model (see Table 3). The two-group hypothesized unconstrained model (Two-Group Model 1) was compared with the nested models. The Two-Group Model 2 had three pathways constrained to be equal across groups. These were the pathways that were significant in the one-group model analyses (from T1 exhaustion to self-efficacy change, from self-efficacy change to T2 disengagement, and from T1 exhaustion to T2 disengagement). These pathways were constrained to be equal (Model 2). In the next nested model (Two-Group Model 3), all structural covariances were constrained to be equal. Finally, the residuals of disengagement at T2 and residuals of self-efficacy change indices were constrained to be equal in the last nested model (Two-Group Model 4). Results showed that the Two-Group Model 2 and Two-Group Model 4 were not significantly different from the hypothesized model (Two-Group Model 1; see Table 3). Based on these findings, the nested model with significant pathways and residuals constrained to be equal across the two groups (Two-Group Model 5) was compared to the hypothesized model. Results indicated that (Two-Group Model 5) was not significantly different from the hypothesized unconstrained model (Two-Group Model 1). Thus, the nested model with significant pathways and residuals constrained to be equal across the two groups (i.e., participants of Study 1 and Study 2) may be accepted.

**Table 3:** **Tests of Invariance for the Hypothesized Model between Study 1 and Study 2**.

**Model**	**Model description**	**χ^2^**	**χ^2^/*df***	**NFI**	**Δχ^2^**	**ΔNFI**
Two-Model Group Model 1	Hypothesized model	10.03	1.67	0.984	−	−
Two-Model Group Model 2	Significant pathways constrained to be equal	16.75	1.86	0.974	6.71	0.011
Two-Model Group Model 3	Covariances constrained to be equal	33.54	2.80	.947	23.51^***^	0.037
Two-Model Group Model 4	Residuals constrained to be equal	10.14	1.27	.984	0.11	0.000
Two-Model Group Model 5	Significant pathways and residuals constrained to be equal	16.88	1.53	0.974	6.85	0.011

*The model-data fit for the unconstrained model was acceptable, RMSEA, 0.045 (90% CI [0.000, 0.093]); CFI, 0.993; TLI, 0.967; SRMR, 0.034. The Δχ^2^ indicates a change in a χ^2^ from the modified hypothesized model. A significant Δχ^2^ value indicates that the model was significantly different from the modified hypothesized model. N = 135 for Study 1 and 193 for Study 2*.

*** *p < 0.001*.

#### Test of invariance of associations across subsamples of men and women

Additional analyses aimed at testing invariance of the nested models across subsamples of men and women were conducted. The hypothesized two-group model without constraints was compared with the two-group nested models with constraints, assuming equal effects for both genders. The nested model developed for the test of invariance between Study 1 and Study 2 (cf. Two-Group Model 1) tested the invariance among men and women.

The two-group model with path coefficients constrained to be equal in men and women was not significantly different from the two-group model without constraints, Δχ^2^ = 6.17, *p* = 0.10. In addition, the two-group nested model with residuals constrained to be equal was not significantly different from the two-group model without constraints, Δχ^2^ = 3.84, *p* = 0.15. However, the two-group nested model with covariances constrained to be equal was significantly different from the two-group model without constraints, Δχ^2^ = 20.58, *p* < 0.01. Therefore, the two-group nested model with path coefficients and residuals constrained to be equal was compared to the two-group model without constraints. Results showed that these models were not significantly different, Δχ^2^ = 9.40, *p* = 0.09; thus, the two-group nested model, assuming equal paths and residuals among men and women may be accepted as the final model. The results indicated that pathways in the tested model were similar in the subsamples of men and women.

#### Differences in mean levels of the study variables: comparing study 1 and study 2

The comparisons conducted for data obtained in Studies 1 and 2 indicated that there were significant differences in the mean levels of the study variables (see Table 2). The Polish sample had significantly higher scores for burnout indicators at T1 and T2 than did the U.S. sample. The U.S. sample, in comparison, had significantly higher scores for self-efficacy at T1 and T2, social support at T1 and T2, and indicated more work experience than did the Polish sample.

### Discussion

The results obtained in Study 2 were consistent with the Study 1 findings. Specifically, high levels of exhaustion at T1 led to a larger decline in self-efficacy, which in turn resulted in a higher level of disengagement at T2. Furthermore, the two-group model analyses indicated that the associations between the key investigated variables were similar across Study 1 and Study 2.

## General discussion

The findings obtained in two samples collected in different cultures provide novel evidence for the direction of the relationship between exhaustion and disengagement in the context of change in personal resources. Both samples demonstrate that exhaustion predicted disengagement approximately 6-months later. Additionally, the effects of exhaustion on disengagement were mediated by an index of change in self-efficacy beliefs where higher exhaustion led to a larger decline in self-efficacy across 6 months, which in turn resulted in higher disengagement levels.

The present study confirms the assumptions formulated in the process models advocated by Leiter and Maslach (1988) and Lee and Ashforth (1993). Besides confirming earlier findings (Toppinen-Tanner et al., 2002; Taris et al., 2005; Diestel and Schmidt, 2010) our two-study investigation points out that these associations are invariant (i.e., similar in strength) across two distinct samples of human services workers, differing in terms of country of employment, type of clients (civilian vs. military), or type of occupation. Furthermore, the associations are similar although the levels of burnout components or work experience vary across the samples.

Our investigation attempted to test for the underlying mediating mechanisms which may explain why exhaustion predicts disengagement. Therefore, it goes beyond previous theoretical and empirical approaches that assumed and tested the direct effects of exhaustion on disengagement (Leiter and Maslach, 1988; Lee and Ashforth, 1993; Toppinen-Tanner et al., 2002; Taris et al., 2005; Diestel and Schmidt, 2010). The findings are also in line with meta-analyses indicating that self-efficacy is relatively strongly related to burnout components across occupation groups, countries, and professionals' age and gender (Shoji et al., 2015). In line with the COR theory and the theorized loss spiral (Hobfoll, 1989, 2011), it appears that an individual's state of exhaustion may trigger a decline in personal resources (a negative change in self-efficacy beliefs), which in turn leads to greater disengagement. Thus, besides results obtained by Brouwers and Tomic (2000) that suggested the mediating role of the *levels* of self-efficacy, and in line with COR we indicated that the mediation between exhaustion and disengagement referred to a *change* in self-efficacy. This process offers a much richer appreciation for the coping dynamics involved with burnout. One could argue that the increase in disengagement is a specific coping response to a sense of increasing personal vulnerability comprised of physical fatigue and increasing self-doubt concerning one's capability to manage work related demands.

The effects of exhaustion on disengagement may be further explained by changes in other mediating mechanisms (e.g., personal growth) triggered by stressful events. So far, the mediating roles of evaluations of personal change (or self-evaluations other than self-efficacy) have been addressed in the context of indirect exposure to traumatic material at work (i.e., via traumatized client; cf. Shoji et al., 2014). Future research investigating exhaustion—disengagement association among various types of human services workers could utilize this approach and look for the mediating mechanism of spiral losses/negative changes in self-evaluations and beliefs such as identifying priorities in life or meaning in life (Arnold et al., 2005; Park, 2010).

In contrast to the “resource caravan” hypothesis (Hobfoll, 2011), we did not find that a change in self-efficacy and a change in perceived social support operated in sequence. Furthermore, we did not confirm the cultivation hypothesis, suggesting that self-efficacy prompts social support, which in turn affects workers' well-being (Schwarzer and Knoll, 2007). The effect of exhaustion on disengagement was explained only by self-efficacy changes. Our findings are partially in line with earlier research by Schaufeli et al. (2009). They found no evidence for the existence of a loss cycle that included social support loss (Schaufeli et al., 2009).

A lack of effect of social support on disengagement may result from the fact that this variable operates indirectly, via other resources. For example, social support may directly affect perceived personal growth (Shoji et al., 2014) and perceived personal growth may in turn be directly related to well-being outcomes. Thus, social support might have a potential to contribute to a spiral gain of other resources, reducing disengagement. Future, studies need to investigate if social support may operate in concert with other stress-related cognitions, contributing either to spiral loss or spiral gain of resources.

Burnout and personal resources are relatively stable. Longitudinal research conducted over periods ranging from 4 months to 7 years indicated that approximately one-third of variance of burnout and about a half of variance of resources may be stable over years (for overview see Seppälä et al., 2015). Studies 1 and 2 applied relatively short follow-ups, but stability of analyzed constructs was similar to stability found in earlier research (Seppälä et al., 2015). The relatively high stability of resources and burnout may reduce the likelihood of finding the effects of change of resources (such as social support) on burnout components.

The present study has its limitations. Our approach to burnout focuses on its two dimensions, which are included into some but not all burnout models (cf., Melamed et al., 2006). Therefore, any conclusions referring to the internal structure of burnout should be treated with caution and not generalized beyond exhaustion and disengagement. We controlled for the number of years of work experience and observed the effects of work experience similar to those found in earlier research (for meta-analysis see Brewer and Shapard, 2004). However, we did not control for other potential confounders, such as job demands, job control, or other indicators of job stress.

Although, both of our studies were longitudinal, there were only two measurement points. A four-wave investigation would be optimal to test a sequential multiple mediation model with two mediators and we plan to conduct this type of investigation next. Regarding a methodological limitation related to a longitudinal design, the research procedures did not allow us to explain reasons for dropouts at T2. Relatively high attrition rates limit the generalizability of the findings. Although for a majority of variables we found no systematic dropout patterns, we observed trends indicating a systematic character of dropout for two variables in Study 1 and one variable in Study 2. In Study 1, participants with high disengagement (T1) were lost at the follow-up. Therefore, the findings of Study 1 may better reflect the effects observed for those whose burnout was lower at T1. Importantly, the findings of Study 1 and Study 2 revealed similar patterns of associations, and there was no systematic dropout for burnout indicators in Study 2.

Another limitation refers to the choice of self-efficacy measure. Although our findings suggested that the scale had good reliability and shared less than 38% of variance with other constructs, confirming its discriminant validity, future research testing the validity of the burnout self-efficacy scale are needed.

The study is also limited in that we tested only one direction from exhaustion to disengagement, which is in line with previous findings (e.g., Toppinen-Tanner et al., 2002; Taris et al., 2005; Diestel and Schmidt, 2010). Testing competing models could provide additional conclusions, however, the best test for the directions of the relations between exhaustion and disengagement could be obtained in multi-wave natural experiment studies observing workers from the first days of their employment throughout their professional career. Furthermore, we did not test competing hypotheses, such as job resources (e.g., self-efficacy and social support) predicting job burnout components or that job burnout components could explain job resources. Longitudinal studies carried over several years indicate that these relationships may be bidirectional (Seppälä et al., 2015). Future, research should further investigate the directions of the relationships among burnout components, self-efficacy, and social support.

Finally, in line with earlier findings (e.g., Shoji et al., 2014), we only tested the cultivation hypothesis. Future, research should consider alternative models to understand how self-efficacy and other personal resources (e.g., perceived social support) and environmental conditions (e.g., work related constraints) may interact when explaining burnout components. Accounting for other resources referring to social environments or self-beliefs would enrich our understanding of the mechanisms explaining how exhaustion influences disengagement.

In sum, this is the first longitudinal two-study cross-cultural investigation on how changes in personal resources mediate between exhaustion and disengagement, measured 6 months apart. Both studies consistently indicate that reductions in job burnout self-efficacy were determined by exhaustion and facilitated greater disengagement. Future research that includes the intersection of personal resources and environmental factors in untangling the negative components of burnout will help move this literature forward informing critical interventions. In particular, the findings may have some implications for prevention of the escalation of burnout. Interventions aiming at a reduction of negative consequences of work stress may target workers with higher levels of exhaustion and work to enhance their self-efficacy beliefs specifically related to the negative consequences of work stress.

## Author contributions

CB, RC served as the P.I.'s on the project providing significant conceptual and design contribution and significantly contributing to drafting the manuscript. CB, RC, AR, CY were involved in data acquisition. KS, RC, AL, were involved in the statistical analyses for the projects. CB, RC, KS, AR, CY, AL AK were involved in manuscript preparation and final approval of the paper. CB, RC, KS, AR, CY, AL, AK agree to be accountable for all aspects of the work specifically to responding to questions related to the accuracy or integrity of any part of the work.

## Funding

This research was supported in part by a research grant to Charles Benight awarded and administered by the U.S. Army Medical Research & Materiel Command (USAMRMC) and the Telemedicine & Advanced Technology Research Center (TATRC) at Fort Detrick, MD under Contract Number W81XWH-11-2-0153 and a research grant N N106 139537 from the Polish National Science Center awarded to Roman Cieslak. The contribution of Aleksandra Luszczynska is supported by the Foundation for Polish Science, Master program.

### Conflict of interest statement

The authors declare that the research was conducted in the absence of any commercial or financial relationships that could be construed as a potential conflict of interest.
